# HCV genotyping using statistical classification approach

**DOI:** 10.1186/1423-0127-16-62

**Published:** 2009-07-08

**Authors:** Ping Qiu, Xiao-Yan Cai, Wei Ding, Qing Zhang, Ellie D Norris, Jonathan R Greene

**Affiliations:** 1 Molecular Design and Informatics, Schering-Plough Research Institute, 2015 Galloping Hill Road, Kenilworth, NJ 07033, USA; 2 Biotechnology and Molecular Bioanalytics, Schering-Plough Research Institute, 1011 Morris Avenue, Union, New Jersey 07083, USA

## Abstract

The genotype of Hepatitis C Virus (HCV) strains is an important determinant of the severity and aggressiveness of liver infection as well as patient response to antiviral therapy. Fast and accurate determination of viral genotype could provide direction in the clinical management of patients with chronic HCV infections. Using publicly available HCV nucleotide sequences, we built a global Position Weight Matrix (PWM) for the HCV genome. Based on the PWM, a set of genotype specific nucleotide sequence "signatures" were selected from the 5' NCR, CORE, E1, and NS5B regions of the HCV genome. We evaluated the predictive power of these signatures for predicting the most common HCV genotypes and subtypes. We observed that nucleotide sequence signatures selected from NS5B and E1 regions generally demonstrated stronger discriminant power in differentiating major HCV genotypes and subtypes than that from 5' NCR and CORE regions. Two discriminant methods were used to build predictive models. Through 10 fold cross validation, over 99% prediction accuracy was achieved using both support vector machine (SVM) and random forest based classification methods in a dataset of 1134 sequences for NS5B and 947 sequences for E1. Prediction accuracy for each genotype is also reported.

## Background

Hepatitis C virus has a positive-sense single-stranded RNA genome of about 9.6 kb containing one long open reading frame (ORF) with untranslated regions at both ends [1]. The polyprotein is processed into structural and nonstructural proteins. The core and the two envelope proteins (E1 and E2) are part of the virion. So far, six major genotypes (HCV-1 to HCV-6) have been described, each containing multiple subtypes (e.g., 1a, 1b, etc.). The isolates formerly published as genotypes 7 to 11 are now considered subtypes within genotypes 3 (genotype10) and 6 (genotype 7, 8, 9, and 11) [2,3].

Infection by HCV is the leading cause of chronic liver disease worldwide [4]. The overall prevalence of HCV infection in the United States is 1.8%, with most of the patients unaware of their infection and risk for developing cirrhosis and hepatocellular carcinoma [5]. The most prevalent genotypes in the U.S. were 1 (71.0%), 2 (14.3%), and 3 (11.6%), followed by less common types 4 (1.7%), and 6 (1.5%). The remaining types represent less than 1% of the population. The prevalence of each genotype in the population was relatively stable [6].

The genotype of the HCV strain appears to be an important determinant of the severity and aggressiveness of liver infection, as well as patient response to antiviral therapy [7]. HCV genotypes display significant differences in their global distribution and prevalence, making genotyping a useful method for determining the source of HCV transmission in an infected localized population. Due to the chronic nature of HCV infection and the tremendous burden on healthcare resources, clinicians and researchers have looked for key epidemiological, pathological and viral characteristics that may provide insight into disease progression, severity and response to therapy to permit the administration of effective therapeutic regimens as well as long-term management of infected individuals [8]. The best available therapy for HCV infection, interferon in combination with ribavirin, is effective in only a subset of cases. The sustained virologic response rates of treated patients range from 30 to 70% and are dependent on several key clinical and virologic factors [9,10]. Genotype 1 infection has the lowest response rates and requires the longest therapy [11]. The HCV genotype has emerged as an important factor both in predicting a sustained response to and in determining the duration of antiviral therapy.

Quick and accurate genotyping of hepatitis C virus (HCV) is becoming increasingly important for clinical management of chronic infection and as an epidemiological marker [12]. Several methods for genotyping HCV have been developed, including direct DNA sequencing [13,14], type specific PCR [15], restriction fragment length polymorphism, line probe assays [16], primer-specific and mispair extension analysis [17], heteroduplex mobility analysis by temperature gradient capillary electrophoresis [18] and denaturing high preference liquid chromatography [19]. Since routine sequence analysis of larger genomic regions is extremely laborious, many laboratories have developed more rapid genotyping methodologies. Crucial to the development of genotyping assays is the choice of the genomic region to be analyzed. The region must contain subtype and type specific motifs which faithfully represent the diversity of the entire genome. In the meantime, variability of the region to be analyzed should be sufficiently low to allow PCR amplification of all HCV genotypes. Several regions of the HCV genome have been analyzed with the purpose of genotypic classification. The 5' NCR, CORE, E1 and NS5B regions have been frequently amplified and studied for the purpose of genotypic classification [3,20,21] with NS5B more often used for differentiation of subtypes and confirmation of genotyping results in research settings.

Despite the limited sequence diversity found within the HCV 5' NC region (NCR), practical considerations have made the 5' NCR the preferred target for HCV genotyping in most diagnostic laboratories [21]. Several HCV genotyping assays are currently commercially available, including the TRUGENE HCV 5'NC genotyping kit (TRUGENE 5'NC; Bayer HealthCare LLC, Berkeley, Calif.) and the VERSANT HCV genotype assay (LiPA; Bayer HealthCare LLC). However, these methods were often found not to be definitive, as more sequence data became available suggesting 5' NCR might not contain enough sequence characteristics that can be used to differentiate all genotypes and subtypes [22].

A systematic comparison of the common HCV genome regions in terms of their ability to predict viral genotype is not currently available. In this study, utilizing the HCV sequence records retrieved from GenBank, conservation analysis of each position of the HCV genome within each genotype was performed. Classification models were built based on nucleotide signatures selected from four HCV regions to differentiate 10 major genotypes and subtypes. Two modern statistical classification methods were evaluated in this paper: support vector machine (SVM) and random forest.

## Methods

### Databases and resources

GenBank Release 149 was downloaded from [23]. ClustalW [24] was used for multiple sequence alignments. All statistical analyses were carried out with R using packages randomForest (from A. Liaw and M. Wiener) for random forest and e1071 (E. Dimitriadou, K. Hornik, F. Leisch, D. Meyer and A. Weingessel) for SVM. All non-commercial software used in this study was written in PERL 5.0.

### Construction of alignment

All HCV related sequences were extracted from GenBank (Release 149) [23] by using the keywords HCV or Hepatitis C. In order to reduce weighting bias, redundant sequences that might belong to the same isolates were removed. For any two sequences that shared either 100% identity over 500 bases or 100% identity over the entire length of a 150 to 500 base sequence (excluding the 5' UTR region), the longer sequence was chosen for the dataset. Very short sequences (<150 bases) were excluded from the analysis. D90208 was chosen as the organizing template for its fully annotated genome in GenBank. (Other organizing HCV genomes yielded virtually identical consensus sequences and PWM profiles.). Due to the extreme genetic heterogeneity of the HCV genome and the large number of complete and partial sequences in the public database, a direct genome wide sequence alignment was not feasible. Pairwise alignments were made for all HCV sequences with genotype information (total 10014 sequences) against D90208. Nucleotides at each position were extracted from the alignments. For each position on the HCV genome, nucleotide frequency in the overall HCV population as well as in each genotype was calculated. A global position weight matrix (PWM) was made as described previously [25]. Genotype specific PWMs were also made accordingly. Genome wide PWMs compiled in this step as well as genotype specific PWMs were used to impute missing nucleotides in partial HCV sequences used in model training and in the prediction data set.

### Genotypes and HCV subregions used in this analysis

The most popular genotypes (with at least 40 sequence records in GenBank) were chosen for this study to warrant significant statistical analysis. The genotypes and subtypes used in this study are 1a, 1b, 2a, 2b, 2c, 3a, 3b, 4, 5, and 6. For sequences that belong to rare genotypes [4-6], genotypes were used instead of subtypes for genotype classification. For example, all the 4a and 4b subtype sequences were classified into genotype 4. The objective of this study is to explore the possibility of using a statistical modeling approach in predicting the HCV genotype and to provide direction in choosing the HCV region for genotype classification using a sequencing based approach. Therefore, sub-region sequencing, which can be achieved in one sequencing read experimentally, was preferable (~500 bp). Since most of the HCV sequences retrieved from GenBank are partial sequences, a sub region was selected for each HCV genome region (5' NCR, CORE, E1 and NS5B) in order to balance the sequence coverage of each genotype (Table 1). The total number of sequences which cover each sub region were randomly divided into two equal subsets. One subset was used for model training and model building while the other set was used to estimate the generalization power of the model.

**Table 1:** Sub regions selected for analysis in this study.

Genome Region	Range on D90208	Region Selected	# of Sequences
5' NCR	1–329	73–298	611

CORE	330–889	330–700	498

E1	900–1475	900–1475	947

NS5B	7587–9413	8200–8600	1134

The sub regions were selected to maximize the sequence record coverage of each genotype and the sizes were limited to the length of one sequencing read (~500 bp).

### Position selection (feature selection) and missing value imputation

To maximize the prediction power and minimize the signature position number required for the prediction model, the nucleotide positions in the HCV genome were pre-selected based on their conservation information provided by PWM. We require that the positions included in model building need to be conserved within genotypes and diversified across genotypes. Positions which are at least 80% conserved within the same genotype were chosen in the model training. Positions that are conserved across all genotypes were eliminated from model training.

Most HCV related sequences retrieved from GenBank were partial sequences and some sequences did not have the full coverage for all signature nucleotide positions selected according to the PWM. To facilitate model building, those missing nucleotide positions for each partial sequence were imputed using the consensus nucleotides derived from the PWM. For the training sequence set, the missing nucleotides were imputed using the genotype specific conserved nucleotides. For the prediction sequence set, missing nucleotides were imputed using conserved nucleotides across all genotypes. Partial sequences missing more than one third of the selected positions were eliminated from both the training and prediction sets.

### Classification methods

Various classical and modern statistical methods are available for classification [26]. To discriminate HCV genotypes using the signature nucleotides in different HCV genome regions, two modern classification methods were chosen: support vector machine (SVM) and random forest.

SVM is a learning algorithm which from a set of positively and negatively labeled training vectors learns a classifier that can be used to classify new unlabeled test samples. SVM learns the classifier by mapping the input training samples {x1, . . . , xn} into a possibly high-dimensional feature space and seeking a hyperplane in this space which separates the two types of examples with the largest possible margin, i.e. distance to the nearest points. If the training set is not linearly separable, SVM finds a hyperplane, which optimizes a trade-off between good classification and large margin. [27]. In addition to linear versions of SVMs, they have been extended to nonlinear cases via kernels. We tested linear, polynomial, sigmoid and radial basis kernels with various other parameters. The performance was evaluated using 10-fold cross validation. In this study, we reported our experimental result using the default kernel implemented in package e1071 (radial basis).

Random forest is a classification algorithm developed by Leo Breiman that uses an ensemble of classification trees. It also provides feature importance [28]. Its basic idea is as follows: A forest contains many decision trees, each of which is constructed by instances with randomly sampled features. The prediction is by a majority vote of decision trees. Random forest uses both bagging (bootstrap aggregation), a successful approach for combining unstable learners, and random variable selection for tree building. Each tree is unpruned (grown fully), so as to obtain low-bias trees; at the same time, bagging and random variable selection result in low correlation of the individual trees. The algorithm yields an ensemble that can achieve both low bias and low variance (from averaging over a large ensemble of low-bias, high-variance but low correlation trees).

### Cross-validation

In order to evaluate the generalization power of each of the classification methods and to estimate their prediction capabilities for unknown samples, we used a standard 10-fold cross-validation technique and split the data randomly and repeatedly into training and test sets. The training sets consisted of randomly chosen subsets containing 90% of each class (genotypes); the remaining 10% of the samples from each class were left as test sets. In order to keep computing times reasonable, we reported accuracy and standard deviation estimates over 100 runs. More runs are required if more accurate estimates are desired. We also reported the accuracy of prediction using the prediction set which are never used for model training.

In order to assess the accuracy of prediction methods, we used three measures: sensitivity, specificity and overall accuracy which are defined by







where TP, FP, TN and FN refer to the number of true positives, false positives, true negatives and false negatives, respectively.

## Results and discussion

A large number of HCV related sequences have been deposited in GenBank, making genome wide comparison of different HCV genotypes and subtypes possible. In this report, 10014 full length and partial HCV sequences with genotype and subtype information were extracted from GenBank (Release 149). Similar databases of HCV genome sequences have been constructed by other groups [29,30]. These HCV sequences were classified into 10 major genotypes and subtypes (1a, 1b, 2a, 2b, 2c, 3a, 3b, 4, 5, 6) in this study. For genotypes that were not well-represented, the subtypes were all represented under the genotype. For example, viral subtypes 4a and 4b were combined and represented by genotype 4. For each of the regions that are widely used for HCV genotyping (5' NCR, CORE, E1 and NS5B), a "sub-region" was selected. Sequence coverage for these sub-regions in GenBank was summarized in Table 1. Table 2 detailed the number of HCV sequences used in the study for each genotype. The total pool of HCV sequences was randomly split into two sets. One set of sequences was used to generate a genome wide consensus sequence and Position Weight Matrix (PWM) and was used for statistical modeling for genotype classification. The other set of sequences was used to test the accuracy of genotype prediction models built using the first set of sequences.

**Table 2:** Number of HCV sequences used in the study for each genotype.

Genotype	# of Sequences
1a	1667

1b	5845

2a	198

2b	406

2c	222

3a	591

3b	168

4	542

5	148

6	227

Intuitively, a good feature set for classification model building should consist of those members highly correlated within a class but uncorrelated with other classes [31]. Finding the "best" set of features to build a predictive model is a complex combinatorial problem and available methods are generally classified into two categories: filtering methods (those which rank individual features according to some criteria) and more involved wrapper algorithms, which use classification methods directly to evaluate a particular set of features. In this study we reported only filtering based methods since they performed reasonably well. We used filtering based variable/feature selection methods using global genome conservation data derived from our PWM. The criteria imposed are that selected signature nucleotide positions need to be at least 80% conserved within genotypes and diversified across genotypes. Partial sequences with missing signature nucleotide sequences were imputed using the PWM we constructed to allow inclusion in the analysis.

Support vector machine (SVM) and random forest are two modern statistical classification methods. Classification based on SVMs has several applications in bioinformatics and computational biology. It has been widely used to predict protein secondary structures [32]; protein-protein binding site [33,34]; remote protein homologs [35]; protein domains [36]; protein subcellular localization [37,38] and gene and tissue classification from microarray expression data [39]. Random forest is relatively new and comparisons with SVM have not been widely reported.

**Figure 1:**
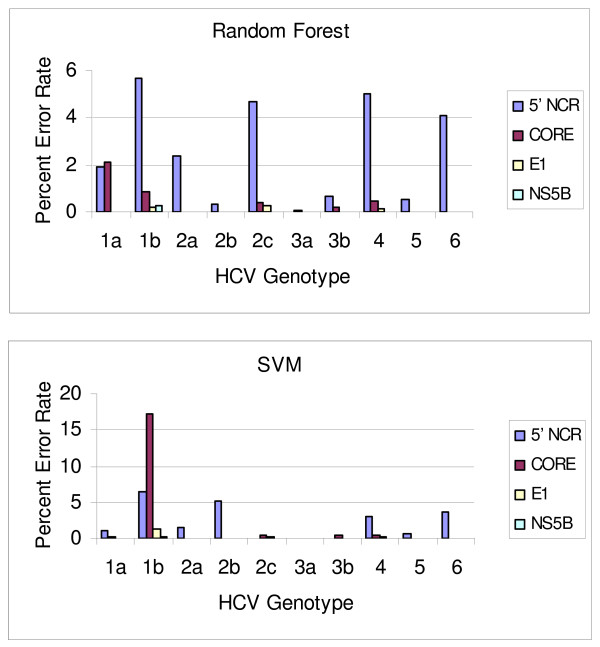
**Average classification error rate (percent) over 100 runs on different genotypes from 10-fold cross-validation**.

We generated SVM and random forest models for features (nucleotide positions) selected from four HCV regions (5' NCR, CORE, E1 and NS5B) to predict the most common HCV genotypes and subtypes. Error rates are computed as average error rates over 100 runs of 10-fold cross validation, that is, a cross-validation procedure of training on 90% of the data and testing on the remaining 10% was repeated 100 times and the errors averaged (Table 3). Both SVM and random forest methods demonstrated comparable predictive power in this study. However, the random forest method seems to perform slightly better. Error rates for each genotype and subtype were also estimated for both SVM and random forest models (Figure 1). Notably, predictive models derived from features selected from the NS5B and E1 regions tended to have more predictive power than those from more conserved regions such as 5' NCR and CORE. This was observed for all genotypes (Figure 1). Traditionally, the conserved nature of the 5'NCR has made it the preferred target for HCV RNA detection tests, and sequence analysis of amplicons from these tests is the most efficient way to genotype HCV in a clinical laboratory setting since both tests can be completed with the product from a single amplification reaction. However, as indicated in this study, 5' NCR might not be the best choice if more accurate genotyping results are required. This observation is in accordance with a previous study which showed that 5'NCR is too conserved for accurate discrimination of all subtypes [40-42].

**Table 3:** Average error rates over 100 runs on features from four HCV genome regions using two different classification algorithms.

Classification Method	Region on HCV Genome			
	
	5' NCR	CORE	E1	NS5B
SVM	21.98	19.66	1.60	0.21

Random Forest	24.28	3.98	0.56	0.19

Error rates are computed as average error rates over 100 runs, that is, a cross-validation procedure of training on 90% of the data and testing on the remaining 10% was repeated 100 times and the errors averaged.

The average conservation scores for the selected regions in 5' NCR, CORE, E1, NS5B are 96%, 91%, 80% and 80% respectively suggesting that a region which can serve to discriminate genotypes tends to be modestly conserved if not the least conserved. Practically, it is considerably easier to develop assays for more conserved regions such as 5' NCR. However, with the HCV global PWM in hand, it is straightforward to derive the most conserved sequence stretches within NS5B and E1 which facilitates the design of robust nucleotide primers. This process and associated criteria have been described in our previous study [25]. Genotype or subtype specific primers with higher selectivity for NS5B and E1 can also be derived from PWM if necessary.

As indicated in Figure 1, the error rate for determining subtype 1b is the most significant contributor to the overall error rate, especially in models built on the 5' NCR. This might be caused by the high degree of genome similarity between subtype 1a and 1b. The consensus sequences of 1a and 1b share over 99% similarity in 5' NCR (73–298); 95% in CORE (330–700); 76% in E1 (900–1475); 83% in NS5B(8200–8600) respectively. In models built using NS5B or E1 signature nucleotides, genotypes 1a and 1b can be easily differentiated with very low error rate suggesting that closely related subtypes can be effectively differentiated by using a less conserved region. The cause of the small remaining error rate is not very clear and one possible source might be misclassified records from GenBank that were included in the model building and prediction data set. Manual inspection of some of the mispredicted records indicated that at least some of them are due to the short available sequences and a significant amount of data imputation for signature nucleotide positions.

The predictive accuracy of SVM and random forest model for region NS5B and E1 on unseen HCV sequences (as described in Materials and Methods) are also very good (Table 4), with accuracy in the high ninety percent range. Analyses of the misclassification cases also suggests that sequencing more than one region, predicting with more than one model, and taking majority vote will give maximal predictive accuracy (data not shown).

**Table 4:** HCV genotype prediction accuracy using an independent data set (result was reported for models built based on NS5B and E1 only)

Genotype	E1						NS5B					
	SVM			RF			SVM			RF		

	SN	SP	AC	SN	SP	AC	SN	SP	AC	SN	SP	AC

1a	98.9	98.3	98.8	98.4	96.7	97.4	100	100	100	100	100	100

1b	94.8	99.7	98.8	100	99.7	98.2	99.4	100	99.8	99.4	99.3	99.3

2a	100	100	100	100	100	100	100	100	100	75	100	99.8

2b	100	100	100	100	100	100	100	100	100	100	100	100

2c	100	100	100	55.6	99.8	99	100	100	100	93.3	100	99.8

3a	100	100	100	100	100	100	100	99.8	99.8	100	99.8	99.9

3b	100	100	100	100	100	100	100	100	100	100	100	100

4	100	99.8	99.8	90.4	100	99	100	100	100	100	100	100

5	100	100	100	100	100	100	96.3	100	99.8	96.3	100	99.8

6	100	100	100	84.6	100	98.4	100	99.8	99.8	80	100	99.8

The predictive performance of models built on the selected variables using a recursive redundant variable removal approach was also examined. The predictive accuracy of the models after backward feature elimination is comparable to that of using signature nucleotides that was selected with a filtering based method (data not shown). Since the goal of this study is to classify HCV genotypes and subtypes, selecting the smallest possible set of features is not the main interest as long as the features can be obtained within one experiment. On the other hand, with all the features being easily obtained within one sequencing read, keeping redundant variables might be beneficial when nucleotide reads at certain positions are not easily available due to experimental reasons.

In conclusion, we have developed SVM and random forest based methods for discriminating HCV genotypes and subtypes. Models built based on features from NS5B and E1 perform better than those based on features from CORE and 5' NCR. In addition, a global PWM for the HCV genome can be used to successfully design both global and genotype and subtype specific primers for less conserved regions such as NS5B and E1 (Table 5).

**Table 5:** Suggested primer stretches (for sequencing and PCR) based on HCV whole genome PWM for analyzing signature nucleotides selected for NS5B and E1 region.

	Forward Primers				Reverse Primers			
	Start	End	Conservation Score (%)	Sequence	Start	End	Conservation Score (%)	Sequence

NS5B	8050	8074	93.2	AGCCAGCTCGCCTTATCGTATTCCC	8629	8605	94.5	GCGGAATACCTGGTCATAGCCTCCG
	
	8083	8107	89.1	GGGTTCGTGTGTGCGAGAAGATGGC	8800	8776	91.1	ACTGGAGTGTGTCTAGCTGTCTCCC
	
	8082	8106	89.0	GGGGTTCGTGTGTGCGAGAAGATGG	8634	8610	89.7	GGGGGGCGGAATACCTGGTCATAGC
	
	8125	8149	85.9	CCACCCTTCCTCAGGCCGTGATGGG	8633	8609	89.7	GGGGGCGGAATACCTGGTCATAGCC
	
	8124	8148	84.3	TCCACCCTTCCTCAGGCCGTGATGG				

								

								

E1	709	733	94.1	CATGCGGCTTCGCCGACCTCATGGG	1612	1588	89.3	TTCAGGGCAGTCCTGTTGATGTGCC
	
	708	732	94.0	ACATGCGGCTTCGCCGACCTCATGG	1605	1581	89.3	CAGTCCTGTTGATGTGCCAGCTGCC
	
	733	757	93.0	GGTACATTCCGCTCGTCGGCGCCCC	1629	1605	83.2	TGAGGCTGTCATTGCAGTTCAGGGC
	
	821	845	91.2	TGCAACAGGGAACCTTCCTGGTTGC				

To ensure optimal polymerization, the 3' end and the penultimate position were required to be G or C with frequencies of ≥0.98 and the upstream position, (3' -2), a G or C with a frequency of ≥0.90 or alternatively an A or T with a frequency of ≥0.95.

## Competing interests

The authors declare that they have no competing interests.

## Authors' contributions

PQ designed the study, carried out the data analysis and drafted the manuscript. WD, EN participated in the data analysis and study design. XYC, QZ, JG participated in the study design and manuscript revising.

## References

[B1] Choo QL, Kuo G, Weiner AJ, Overby LR, Bradley DW, Houghton M (1989). Isolation of a cDNA clone derived from a bloodborne non-A, non-B hepatitis genome. Science.

[B2] Tokita H, Okamoto H, Luengrojanakul P, Vareesangthip K, Chainuvati T, Iizuka H, Tsuda F, Miyakawa Y, Mayumi M (1995). Hepatitis C virus variants from Thailand classifiable into five novel genotypes in the sixth (6b), seventh (7c, 7d) and ninth (9b, 9c) major genetic groups. J Gen Virol.

[B3] Sandres-Saune K, Deny P, Pasquier C, Thibaut V, Duverlie G, Izopet J (2003). Determining hepatitis C genotype by analyzing the sequence of the NS5b region. J Virol Methods.

[B4] Pearlman BL (2004). Hepatitis C treatment update. Am J Med.

[B5] McHutchison JG (2004). Understanding hepatitis C. Am J Manag Care.

[B6] Hoofnagle JH, Wahed AS, Brown RS, Howell CD, Belle SH;, Virahep-C Study Group (2009). Early changes in hepatitis C virus (HCV) levels in response to peginterferon and ribavirin treatment in patients with chronic HCV genotype 1 infection. J Infect Dis.

[B7] Zein NN (2000). Clinical significance of hepatitis C virus genotypes. Clin Microbiol Rev.

[B8] Hnatyszyn HJ (2005). Chronic hepatitis C and genotyping: the clinical significance of determining HCV genotypes. Antivir Ther.

[B9] McHutchinson JG, Gordon SC, Schiff ER, Shiffman ML, Lee WM, Rustgi VK, Goodman ZD, Ling MH, Cort S, Albrecht JK, for The Hepatitis Interventional Therapy Group (1998). Interferon alfa-2b alone or in combination with ribavirin as initial treatment for chronic hepatitis C. N Engl J Med.

[B10] Poynard T, Marcellin P, Lee SS, Niederau C, Minuk GS, Ideo G, Bain V, Heathcote J, Zeuzem S, Trepo C, Albrecht J, for The International Hepatitis Interventional Therapy Group (1998). Randomised trial of interferon α2b plus ribavirin for 48 weeks or for 24 weeks versus interferon α2b plus placebo for 48 weeks for treatment of chronic infection with hepatitis C virus. Lancet.

[B11] Poynard T, McHutchison JG, Goodman ZD, Ling MH, Albrecht J, for The ALGOVIRC Project Group (2000). Is an "a la carte" combination interferon alfa-2b plus ribavirin regimen possible for the first line treatment in patients with chronic hepatitis C. Hepatology.

[B12] Antonishyn NA, Ast VM, McDonald RR, Chaudhary RK, Lin L, Andonov AP, Horsman GB (2005). Rapid genotyping of hepatitis C virus by primer-specific extension analysis. J Clin Microbiol.

[B13] Bukh J, Purcell RH, Miller RH (1992). Sequence analysis of the 5' noncoding region of hepatitis C virus. Proc Natl Acad Sci USA.

[B14] Germer JJ, Majewski DW, Rosser M, Thompson A, Mitchell PS, Smith TF, Elagin S, Yao JDC (2003). Evaluation of the TRUGENE HCV *5'NC *genotyping kit with the new GeneLibrarian module 3.1.2 for genotyping of hepatitis C virus from clinical specimens. J Clin Microbiol.

[B15] Okamoto H, Sugiyama Y, Okada S, Kurai K, Akahane Y, Sugai Y, Tanaka T, Sato K, Tsuda F, Miyakawa Y (1992). Typing hepatitis C virus by polymerase chain reaction with type-specific primers: application to clinical surveys and tracing infectious sources. J Gen Virol.

[B16] Nakao T, Enomoto N, Takada N, Takada A, Date T (1991). Typing of hepatitis C virus genomes by restriction fragment length polymorphism. J Gen Virol.

[B17] Hu YW, Balaskas E, Furione M, Yen PH, Kessler G, Scalia V, Chui L, Sher G (2000). Comparison and application of a novel genotyping method, semiautomated primer-specific and mispair extension analysis, and four other genotyping assays for detection of hepatitis C virus mixed-genotype infections. J Clin Microbiol.

[B18] Margraf RL, Erali M, Liew M, Wittwer CT (2004). Genotyping hepatitis C virus by heteroduplex mobility analysis using temperature gradient capillary electrophoresis. J Clin Microbiol.

[B19] Liew M, Erali M, Page S, Hillyard D, Wittwer C (2004). Hepatitis C genotyping by denaturing high-performance liquid chromatography. J Clin Microbiol.

[B20] Corbet S, Bukh J, Heinsen A, Fomsgaard A (2003). Hepatitis C virus subtyping by a core-envelope 1-based reverse transcriptase PCR assay with sequencing and its use in determining subtype distribution among Danish patients. J Clin Microbiol.

[B21] Nolte FS, Green AM, Fiebelkorn KR, Caliendo AM, Sturchio C, Grunwald A, Healym M (2003). Clinical evaluation of two methods for genotyping hepatitis C virus based on analysis of the 5' noncoding region. J Clin Microbiol.

[B22] Halfon P, Trimoulet P, Bourliere M, Khiri H, de Ledinghen V, Couzigou P, Feryn JM, Alcaraz P, Renou C, Fleury HJ, Ouzan D (2001). Hepatitis C virus genotyping based on 5' noncoding sequence analysis (Trugene). J Clin Microbiol.

[B23] Benson DA, Karsch-Mizrachi I, Lipmann DJ, Ostell K, Wheeler DL (2006). Genbank. Nucleic Acids Res.

[B24] Thompson JD, Higgins DG, Gibson TJ (1994). CLUSTAL W: improving the sensitivity of progressive multiple sequence alignment through sequence weighting, positions-specific gap penalties and weight matrix choice. Nucleic Acids Res.

[B25] Qiu P, Cai XY, Wang L, Greene JR, Malcolm B (2002). Hepatitis C virus whole genome position weight matrix and robust primer design. BMC Microbiol.

[B26] Khattree R, Naik D (2000). Multivariate Data Reduction and Discrimination with SAS Software.

[B27] Cristianini N, Shawe-Taylor J (2000). An Introduction to Support Vector Machines.

[B28] Breiman L (2001). Random Forests. Machine Learning.

[B29] Kuiken C, Yusem K, Boykin L, Richardson R (2005). The Los Alamos hepatitis C sequence database. Bioinformatics.

[B30] Combet C, Penin F, Geourjon C, Deleage G (2004). HCVDB: hepatitis C virus sequences database. Appl Bioinformatics.

[B31] Hall M (1999). Correlation-based feature selection for machine learning. PhD Thesis.

[B32] Nguyen MN, Rajapakse JC (2005). Two-stage multi-class support vector machines to protein secondary structure prediction. Pac Symp Biocomput.

[B33] Bradford JR, Westhead DR (2005). Improved prediction of protein-protein binding sites using a support vector machines approach. Bioinformatics.

[B34] Res I, Mihalek I, Lichtarge O (2005). An evolution based classifier for prediction of protein interfaces without using protein structures. Bioinformatics.

[B35] Busuttil S, Abela J, Pace GJ (2004). Support vector machines with profile-based kernels for remote protein homology detection. Genome Inform.

[B36] Vlahovicek K, Kaján L, Agoston V, Pongor S (2005). The SBASE domain sequence resource, release 12: prediction of protein domain-architecture using support vector machines. Nucleic Acids Res.

[B37] Hua S, Sun Z (2001). Support vector machine approach for protein subcellular localization prediction. Bioinformatics.

[B38] Nair R, Rost B (2005). Mimicking cellular sorting improves prediction of subcellular localization. J Mol Biol.

[B39] Brown MP, Grundy WN, Lin D, Cristianini N, Sugnet CW, Furey TS, Ares M, Haussler D (2000). Knowledge-based analysis of microarray gene expression data by using support vector machines. Proc Natl Acad Sci USA.

[B40] Smith DB, Mellor J, Jarvis LM, Davidson F, Kolberg J, Urdea M, Yap PL, Simmonds P (1995). Variation of the hepatitis C virus 5' noncoding region: implications for secondary structure, virus detection, and typing. J Gen Virol.

[B41] Chen Z, Weck KE (2002). Hepatitis C virus genotyping: interrogation of the 5' untranslated region cannot accurately distinguish genotypes 1a and 1b. J Clin Microbiol.

[B42] Laperche S, Lunel F, Izopet J, Alain S, Deny P, Duverlie G, Gaudy C, Pawlotsky JM, Plantier JC, Pozzetto B, Thibault V, Tosetti F, Lefrere JJ (2005). Comparison of Hepatitis C Virus NS5b and 5' Noncoding Gene Sequencing Methods in a Multicenter Study. J Clin Microbiol.

